# Prevailing Outcome Themes Reported by People With Degenerative Cervical Myelopathy: Focus Group Study

**DOI:** 10.2196/18732

**Published:** 2021-02-03

**Authors:** Danyal Zaman Khan, Siobhan Mairead Fitzpatrick, Bryn Hilton, Angus GK McNair, Ellen Sarewitz, Benjamin Marshall Davies, Mark RN Kotter, AO Spine Knowledge Forum Spinal Cord Injury

**Affiliations:** 1 Academic Neurosurgery Department University of Cambridge Cambridge United Kingdom; 2 Department of Psychology University of Warwick Coventry United Kingdom; 3 Colchester Hospital University East Suffolk and North Essex NHS Foundation Trust Colchester United Kingdom; 4 Centre for Surgical Research Bristol Medical School: Population Health Sciences University of Bristol Bristol United Kingdom; 5 Goffin Consultancy Cambridge United Kingdom; 6 Wellcome Trust & MRC Cambridge Stem Cell Institute University of Cambridge Cambridge United Kingdom; 7 AO Spine Davos Switzerland

**Keywords:** cervical, myelopathy, spondylosis, spondylotic, stenosis, disc herniation, ossification posterior longitudinal ligament, qualitative, thematic analysis, core outcomes set, consensus, Delphi, patient perspectives

## Abstract

**Background:**

Degenerative cervical myelopathy (DCM) arises when arthritic changes of the cervical spine cause compression and a progressive injury to the spinal cord. It is common and potentially disabling. People with DCM have among the lowest quality of life scores (Short Form Health Survey–36 item [SF-36]) of chronic disease, although the drivers of the imapact of DCM are not entirely understood. DCM research faces a number of challenges, including the heterogeneous reporting of study data. The AO Spine Research Objectives and Common Data Elements for Degenerative Cervical Myelopathy (RECODE-DCM) project is an international consensus process that aims to improve research efficiency through formation of a core outcome set (COS). A key part of COS development process is organizing outcomes into domains that represent key aspects of the disease. To facilitate this, we sought to qualitatively explore the context and impact of patient-reported outcomes in DCM on study participants.

**Objective:**

The goal of the research was to qualitatively explore the patient-reported outcomes in DCM to improve understanding of patient perspective and assist the organization of outcomes into domains for the consensus process.

**Methods:**

Focus group sessions were hosted in collaboration with Myelopathy.org, a charity and support group for people with DCM. A 40-minute session was audiorecorded and transcribed verbatim. Two authors familiarized themselves with the data and then performed data coding independently. Codes were grouped into themes and a thematic analysis was performed guided by Braun and Clarke’s 6-phase approach. The themes were subsequently reviewed with an independent stakeholder with DCM, assisting in the process of capturing the true context and importance of themes.

**Results:**

Five people with DCM (3 men and 2 women) participated in the focus group session. The median age was 53 years, and the median score on the modified Japanese Orthopaedic Association scale was 11 (interquartile range 9.5-11.5), indicating the participants had moderate to severe DCM. A total of 54 codes were reviewed and grouped into 10 potential themes that captured the impact of the disability on people with DCM: acceptance of symptoms, anticipatory anxiety, coping mechanisms/resilience, feelings of helplessness, financial consequences, lack of recognition, mental health impact, loss of life control, social reclusiveness and isolation, and social stigma.

**Conclusions:**

This qualitative analysis of the perspectives of people with DCM has highlighted a number of prevailing themes currently unmeasured in clinical research or care. The determinants of low quality of life in DCM are currently unknown, and these findings provide a novel and so far, unique perspective. Continued inclusion of online communities and use of targeted digital software will be important in establishing a consensus-based COS for patients with DCM that is inclusive of all relevant stakeholders including people with DCM.

## Introduction

Degenerative cervical myelopathy (DCM) arises when arthritic changes of the cervical spine cause compression and a progressive injury to the spinal cord [1]. This is the most common cause of spinal cord dysfunction worldwide [2]. Sadly, most patients are left with life-changing disability, despite treatment. A recent study has shown people with DCM to have among the lowest quality of life scores (Short Form Health Survey–36 item [SF-36]) of chronic disease, although the drivers for this are not entirely understood [3,4]. Research advances are clearly required.

Currently, however, DCM research faces a number of challenges [5]. This includes the heterogeneous reporting of study data, making it difficult to synthesize or compare research [3,6]. AO Spine Research Objectives and Common Data Elements for Degenerative Cervical Myelopathy (AO Spine RECODE-DCM) is an international initiative in response to this to provide tools that can support research progress [7]. This includes the formation of a core outcome set (COS).

COS development starts with the development of a long list of outcomes that is put through a consensus process to decide which outcomes are most important and should be included in the COS [8]. An important aspect of forming COS is to ensure representation among all stakeholder groups, including those living with the condition. This latter aspect is argued to be essential to supporting meaningful research [9]. This is exemplified in DCM by the recent valuation of people with DCM recovery priorities [10], which identified pain as the overall priority despite its infrequent representation in DCM research [3,6].

There are a number of different methods for involving people with DCM in the formation of a long list of outcomes [8,11]. One is to undertake qualitative interviews and undertake content analysis, which we have recently performed [12]. One of the limitations of this method is it fails to measure the significance or context of an outcome. While this will be mitigated during the Delphi survey, the information would be helpful to support the rationing of an outcome list (to reduce the number of outcomes listed in a survey and respondent fatigue) and the formation of domains (categories of outcomes). The selection of domains is an important step, as each category will expect representation and therefore it should reflect a key aspect of the disease [11].

Thus, the purpose of this study was to qualitatively explore descriptions of people with DCM and the impact of DCM on their lives to identify prevailing themes and their significance. These themes could aid our understanding of the perspective of people with DCM and assist the organization of outcomes into domains for a Delphi process.

## Methods

### Overview

The objective of this process was to explore the individual impact of the outcomes reported by people with DCM using qualitative analysis techniques. More specifically, we sought to understand the context and implications of DCM outcomes on the psychosocial aspects of the lives of people with DCM. We referred to the Consolidated Criteria for Reporting Qualitative Research (COREQ) checklist for guidance throughout [13] (Multimedia Appendix 1).

### Focus Group Workshops

Attendees of a Patient and Public Involvement Day at Cambridge University Hospital on September 21, 2017 hosted by Myelopathy.org, a charity and support group for people with DCM, were invited to participate in a focus group. Convenience sampling was employed, with the event advertised to registered members of Myelopathy.org. As previously described, this group consisted of DCM patients and their supporters [12]. A focus group style was chosen, facilitating in-depth insight into participant perspectives through principles such as social constructivism [8,14].

Focus groups were initially conducted as separate stakeholder groups (supporters and people with DCM), during which field notes of potential outcomes were generated (reported elsewhere [12]). This list was used to guide the main focus group session consisting of both supporters and people with DCM. During this main session, an initial open question—“How does DCM affect you?”—was posed, followed by discussion focusing on outcomes and their significance. This discussion concentrated on the experiences of people with DCM and was semistructured, with the interviewers intermittently reorienting dialogue onto the target topic and field notes when necessary (see Topic Guide in Multimedia Appendix 2). The session was halted when data saturation was perceived to have been reached (no further outcomes or outcome impacts emerging from the discussion) [15]. All sessions were facilitated by two interviewers (BMD and MRNK) and recorded for subsequent analysis.

### Ethics and Consent

The survey was granted ethical approval by the University of Cambridge Human Biology Research Ethics Committee with informed consent received from all participants. Participants were briefed on the purpose of the focus group and intended use of the data transcript. All transcripted data was anonymized, and participants were permitted to withdraw at any time.

### Data Analysis

The 40-minute session was audiorecorded and transcribed verbatim (Multimedia Appendix 3) separately by two authors (DZK and SMF) who were not present during the interviews and had no access to any notes taken from the interview. DZK is a medical professional with prior knowledge of qualitative research methods, and SMF is a clinical psychology MSc graduate with qualitative research experience. Any transcription discrepancies were settled by discussion and mutual agreement.

Two authors (DZK and SMF) familiarized themselves with the data and then performed data coding independently. Data coding was done using NVivo software version 10 (QSR International Pty Ltd). Open codes were used (and therefore were not preset and amenable to refinement during the coding process) [16,17]. After independent coding, authors met to discuss codes and group them into themes, a theme being defined as a pattern that captured an interesting or important aspect of the data [16,17]. Thematic analysis using a phenomenological approach was then performed guided by the 6-phase approach of Braun and Clarke [16]. Of note, a predominantly theoretical or top-down approach was adopted, our analysis being driven by the research question [16]. This thematic analysis was performed to a semantic level, identifying themes at the surface level and attempting to understand their significance and context [16,18]. The themes were subsequently reviewed with a stakeholder with DCM (ES), who assisting in the process of capturing the true context and importance of themes.

The above processes produced a list of themes presented as detailed analytical notes. Within these notes, square brackets containing three dots [...] indicate short sections of omitted speech when quoting extracts from the transcript.

## Results

### General

Five people with DCM (3 men and 2 women) attended the focus group session. Three supporters (all women, 2 identifying as partners and 1 as a close friend) accompanied the people with DCM. The median age of participants with DCM was 53 years. One person was awaiting surgery, and 4 participants had undergone surgery for DCM—3 within the last 2 years and 1 over 2 years ago (Table 1). All attendees identified as White. Participants with DCM underwent brief assessment using the modified Japanese Orthopaedic Association scale (mJOA), a validated clinical measure of disease severity scored from 0 to 18. The median mJOA was 11 (interquartile range 9.5 to 11.5), indicating the participants had moderate to severe DCM [19].

**Table 1:** Characteristics of study participants.

Participant number	Age	Gender	Occupation	mJOA^a^	Surgery
1	45	Male	Unemployed^b^	11	ACDF^c^ (C4-C7)
2	54	Female	Unemployed	9	Awaiting
3	65	Male	Retired	11	Yes (unspecified)
4	53	Male	Employed	12	ACDF (unspecified)
5	48	Female	Unemployed^b^	10	ACDF (C6-C7)

^a^mJOA: modified Japanese Orthopaedic Association scale.

^b^Unable to work due to disability.

^c^ACDF: anterior cervical discectomy and fusion.

### Thematic Analysis

Thematic analysis initially yielded 54 codes (Textbox 1), highlighting the self-reported impact of DCM on the lives of participants. These codes were mapped to 10 overarching themes (Textbox 1) that captured the perspectives of the participants on DCM outcomes and their psychosocial impact. The 7 themes felt to be predominant and most frequently expressed during the discussion were explored in detail and presented in the analytical notes below, arranged in no particular order. These raw themes formed the foundations for the refined themes presented in the text.

**Raw themes and constituent codes.**

Diminishing sense of life control:

- Impact on independence and ability to care for self and family
- Inability to plan day to day because of disease unpredictability
- Decreased ability to earn
- Unable to manage pain or disability
- Unable to travel or drive

Feelings of helplessness:

- Irreversible nature of degenerative cervical myelopathy (DCM) is overwhelming
- Progressive disability is overwhelming
- Limited treatment options
- Decreasing autonomy due to disease
- Feelings of needs that are not met by society or health care

Lack of recognition:

- Societal misconceptions and misunderstandings about DCM
- Invisible illness
- Poor appreciation for unpredictability of DCM and symptomology
- Lack of knowledge in general around the debilitating and disabling nature of DCM
- Misconceptions by people with DCM about their disease and its course
- Poor support from welfare services and disability services

Social stigma:

- Lack of recognition of DCM and the resultant disability
- An “invisible” illness
- Misconception of the unpredictability and unreliability of DCM
- Anticipation of stigma from public service workers
- Lack of support from health care professionals

Financial consequences:

- Inability to work
- Difficulty accessing social welfare and disability allowance
- Financial burden of health care
- Accessing private health care for quicker progress
- Debt to compensate for lack of income or support

Social reclusiveness and isolation:

- Disruption to social functioning
- Unpredictability of symptoms affects ability to commit and socialize
- Inability to continue hobbies and pastimes
- Societal stigma and beliefs
- Fear of accidents or incidents
- Unable to travel or drive

Mental health impact:

- Frequent feelings of anxiety and fear about disease
- Feelings of frustration
- Depression
- Suicidal ideation
- Inability to exercise exacerbates mental health difficulties
- Decreased social interaction exacerbates mental health difficulties
- Familial and carer suffering, watching loved ones deteriorate, accessing mental health services
- Feelings of guilt and burdening of carers
- Decreased confidence and self-esteem

Anticipatory anxiety:

- Anxiety from previous experiences of accidents, falls, exacerbation of symptoms affecting activity levels, or ventures*
- Pain avoidance and disability from avoidance
- Unwillingness to leave comfort zones
- Anxiety regarding symptoms makes symptoms worse
- Anxiety regarding future life with disease

Coping mechanisms and resilience:

- Continue day-to-day living despite symptoms
- Commitment to new hobbies
- Working through mental health difficulties
- Persistence despite symptoms and progression

Acceptance of symptoms:

- Accepting chronic nature of symptoms
- Understanding irreversible symptoms
- Adapting lifestyle to symptoms
- Anticipating symptoms and planning for them

### Diminishing Sense of Life Control

DCM had a substantial negative impact on participants’ sense of control, both physically and socially. Impediments on day-to-day living were clear.

One day you can walk, one day you can’t.

Participant 2

Disability affects control over many bodily functions, including bowel and bladder function and sexual function.

Incontinence products...I’m not proud of it but that’s what I have to do!

Participant 5

...having a fulfilling sex life... quite difficult.

Participant 5

Participants spoke of losing their autonomy and the negative impact this has on their psychosocial well-being.

I think we could sum it up in three things here. The frustration, uhhh frustration, depression and physical infirmity.

Participant 2

This understandably affects employment, vocation, and socialization.

You can’t do that physical exercise because you’re physically not fit enough, there’s a big gap in your life.

Participant 2

Daily tasks and activities are contemplated and often impossible to do due to “bad pain management” [Participant 4], meaning psychological protective factors such as exercise, socializing with friends, and family and hobbies see a decline. This extends to working and making a living, consequently “your ability to earn” was a major concern.

If you’re not confident in your finances and what you can provide for your family and that’s a frustration in itself.

Participant 2

Focus group members stressed that this inability to retain control of occupational elements of life was related to feelings of depression.

This is what terrifies me, the fact that I’m going to end up y’know with Tesco online sort of bringing me everything I need and then I’d be so afraid to go out of the house, you’re going to end up clinically depressed.

Participant 2

Additionally, a sense of helplessness was expressed and related to lack of life control due to progressive disease.

I was...shocked when he said “I can’t repair ya.”

Participant 4

There is widespread recognition of the irreversibility of DCM and a paucity of treatment options, which one participant linked to subsequent suicidal thoughts.

When they told me that I wasn’t going to get any better, as I said I was devastated, and I actually ended up feeling suicidal.

Participant 5

An important factor is the receptiveness of society to DCM, which is also believed to influence one’s sense of control. There is a widespread experience of hopelessness in regard to welfare accessibility and entitlement due to the lack of recognition of DCM. Participants mentioned the difficulty they face when undergoing assessment for welfare; the unpredictability of their symptoms and presentations result in inaccurate recognitions of ability and eligibility. One participant linked a positive societal experience regarding her DCM to the familiarity of the disease.

She had a sister-in-law who suffered from cervical myelopathy, so the minute she saw my form she knew exactly what I was talking about and I was fine.

Participant 2

### Lack of Recognition and Social Stigma

As mentioned briefly above, participants described widespread misconceptions among fellow people with DCM, society, and professionals, resulting in further difficulty for those with DCM. For example, some participants themselves were perplexed that the issue itself lay in their neck, particularly when their most noticeable symptoms were elsewhere.

I said “what’s wrong with me neck”...”what you x-raying my neck for, it’s my legs is what’s wrong”

Participant 4

Similarly, societal misunderstanding is a common experience shared by the participants. The spectrum of presentations, day-to-day variability, and misconceptions about DCM mean that people with DCM are regularly being viewed as fine in society regardless of their symptoms.

Yes it looks like I can walk more than 20 meters or more than 50 meters, I can’t do it reliably, I can’t do it repeatedly

Participant 5

The lack of recognition of DCM’s disabling nature is frustrating and distressing.

You get the DWP [Department for Work and Pensions] saying that “oooh you’re not disabled, you can do this you can do that”... I ... I mean I’ve lost my DLA [Disability Living Allowance], I’ve been given zero points for PIP [Personal Independence Payment], I’ve lost my Motorbility Car, I’ve had to buy it, I’ve had to go into more debt than I’m already in

Participant 5

I’ve lost my blue badge! Even a simple thing like a blue badge cos’ the council won’t accept that.

Participant 5

People with DCM receive inadequate support and have no choice but to endure the disabilities or go through lengthy and complicated repeal tribunals.

Yeah it’s a big problem now disability wise, they don’t recognize it do they... I had to fight for mine as well because in 2014 they wouldn’t give me disability at all

Participant 1

Furthermore, this lack of recognition is related to the sense of stigmatization people with DCM experience in society. The relatively invisible nature of the illness was noted to propagate societal misconceptions.

You look perfectly fine, you talk perfectly fine and all of a sudden y’ you’re on your back on the pavement and you can’t get up again...and people think you’re drunk, people think you’re off your face.

Participant 2

You feel like you’re walking out with a... sort of a... a beacon on your head, with the way you move things.

Participant 1

Finally, lack of recognition by health care professionals is a source of feelings of helplessness and frustration.

You feel like banging your head against a brick wall trying to get people to understand you.

Participant 5

Some people with DCM also expressed frustration toward professionals for the follow-up support they’ve received in treating the features secondary to DCM such as depression.

### Financial Consequences

The financial burden of DCM was mentioned in various forms. Inability to work and “your ability to earn” was a major concern.

If you’re not confident in your finances and what you can provide for your family and that’s a frustration in itself and it’s going to make you depressed and angry and down, and that in itself is not good for your general wholesome health.

Participant 2

Moreover, some participants lacked social welfare support.

In 2014 they wouldn’t give me disability at all, because I hadn’t had surgery or anything ... they sent it back saying “no you can’t have it.”

Participant 1

The cost of health care was also mentioned as participants describe long waiting times for diagnostic work-up.

I pay for the scan, just to get it through quicker.

Participant 4

I’m sick of this.

Participant 4

### Social Reclusiveness and Isolation

The multitudinous impacts of DCM culminate in profound social disruption. The day-to-day unpredictability of DCM emerges as a major culprit.

You don’t know until you wake up, whether you’ll be able to walk or not.

Participant 3

You miss social engagements... there is no part of your life that you can say I will definitely be at point A tomorrow or point B tomorrow because you just don’t know.

Participant 2

This is the central core of all these issues, be it finance, family, future, your social circle, you cannot plan. There’s no consistency, one day you can walk, one day you can’t, one day you can travel, one day you can’t.

Participant 2

Inability to socialize results in isolation and loss of community

That’s something that got me down... all my mates are still drinkin’ and so on, so... it puts you y’know the things into a different perspective then.

Participant 1

And when you can’t do that physical exercise because you physically not fit enough, there’s a big gap in your life... what do you fill it with?

Participant 2

### Mental Health Impact

Members of the focus group emphasized the impact DCM has on their mental health, speaking openly about depression, suicidal ideation, anxiety, frustration, and guilt.

It’s going to make you depressed and angry and down, and that in itself is not good for your general wholesome health.

Participant 2

I actually ended up feeling suicidal.

Participant 5

This is intimately related to physical disability.

I was a very physically active person, and it’s very difficult this part of the depressive cycle because when you... part of physical activity makes you feel good, it gives you endorphins, exercise is good for you.

Participant 2

Moreover, guilt was felt as participants placed further reliance on their supporters and their relationships with family members were affected.

It’s had a massive impact on my 11-year-old, ... he’s currently going through CAMHS [Child and Adolescent Mental Health Services] at the moment because he’s struggling ‘cos he’s got now two disabled parents... I might get upset now.

Participant 5

Impacts on mental health appear to act synergistically with physical manifestations of DCM due to its potentially huge life impact and the disability it causes.

### Anticipatory Anxiety

Participants expressed that the anticipation of pain and disability impacted considerably on their daily living. Many expressed anxieties about leaving their home due to fear of falling or exacerbation of pain when attempting to engage in activities of daily living.

Anxious if you’re going to leave your own four walls... you know that oooh I’ve gotta... travel... I’ve gotta do this and it gets your stomach all mmmm...

Participant 3

In fact, anxiety around the symptoms, particularly pain, was emphasized as strongly as the actual pain itself. Pain and discomfort are often consequential to taking control and continuing with daily activities. and participants conveyed the frequent dilemma in deciding whether to sacrifice comfort over control.

So, I either get out there and do them and I run the risk of a fall, which makes me anxious, blood pressure goes up and you’re shaking and that makes simple things difficult. So what’s you’re other option, housebound?

Participant 2

Similarly, this anxiety often aggravates physical symptoms such as shaking, therefore making tasks more difficult and potentially impossible. Understandably, participants referred to it as a vicious cycle.

### Adaptive Coping Mechanisms and Resilience

In spite of the above, people with DCM in the focus group described adjusting lifestyles and taking up new hobbies that were manageable with their symptoms.

I have become...the library’s best customer...I have gone through 6/7 books a week.

Participant 2

Participants were determined to continue with their daily activities and to not succumb to disability.

I force myself to go out, even if it is only to the shops and back.

Participant 2

There is acceptance of symptoms and alterations that inspires the focus group members to be resilient and partake in activities.

I’m just gonna carry on doin’ it because I know I’m going to suffer but I just accept it.

Participant 4

This is supported through active coping mechanisms such as adaptation and self-acceptance, which further ameliorate the effects of DCM on one’s life, allowing for retention of control.

I can tell you where all the toilets are in the town center.

Participant 3

When you’re going out you’re thinking where’s the toilets first of all or start planning.

Participant 4

The strength and power of positive support systems were made apparent by the participants. Many discussed the indirect or subtle motivation that connections such as children, partners, and even pets provided them.

I got a dog to get me out and that’s the reason I go out, not because I have... I should do, but because I have to. I have to obviously with having depression I really struggle to motivate myself.

Participant 5

However, reflecting on these connections revealed feelings of guilt that appeared to be overpowered by the participants’ sheer determination to retain their caregiving roles.

I’ve got the kids to look after, I’ve got dog and bunnies to look after... And so it’s... it’s really important for me to keep...as clear as I can.

Participant 5

## Discussion

### Principal Findings

Qualitative analysis of the perspectives of people with DCM and their supporters has revealed an array of emotive themes relating to the psychosocial impact of living with DCM. We have identified a number of psychosocial implications of DCM, such as loss of control, secondary mental health impacts, external misconception, and anticipatory anxiety and fear. The absence of DCM-specific evidence is in keeping with experience from other chronic disease.

The focus of current DCM literature is on the biological consequences of the disease [3], but psychosocial consequences are also important to people with DCM and their supporters. In a review of reported outcomes in 108 DCM studies, biological outcomes were reported relatively frequently—function was reported in 90% of studies, complications in 52%, imaging in 55%, and pain in 27% of studies [20]. Of note, only 29% of studies reported quality of life (QoL) outcomes, with 80% of these studies using the SF-36 [3]. Although the SF-36 captures, briefly, the impact of depression and anxiety on the physical and social functioning of the individual, it does not capture other emotions, the relationship of people with DCM with the disease, and the social context of DCM (as highlighted in our themes) [21]. Indeed, people with DCM often tend to focus more on the effects of disease on their life (eg, the impact of unpredictable variations in disease) and wider aspects of health, as opposed to the disease process itself [22]. Indeed, there may be discordance between perspectives of researchers and people with DCM on the important outcomes in DCM [3,10]. Studies that have measured the quality of life impact of DCM using the SF-36 show that DCM encompasses among the lowest scores [4]. In the mental component score (covering energy levels, mental health, emotional role functioning, and social functioning), DCM is second only to that of back pain/sciatica [23]. Similarly, DCM scored low on the physical component score (PCS) of SF-36, second only to heart failure [3]. Interestingly, there is discordance between the severity of physical symptoms and general quality of life measures and mental health scores [24], and the determinants of QoL are unknown.

Moreover, exploration of the psychological impact of other chronic illnesses in general adds context to our findings. Anticipatory fear and anxiety is emphasized in the fear-avoidance model of chronic pain [25]. This details the disabling cycle that can occur when an individual experiences pain-related fear. To avoid this fear, individuals become hypervigilant which can lead to further isolation, disability, and often, depression. However, positive coping mechanisms such as acceptance of symptoms (eg, acceptance of chronic pain) predicts positive mental well-being beyond actual severity of pain [26]. Acceptance facilitates more engagement with activities of daily living and may empower feelings of cognitive control over symptoms [26]. Indeed, members of our focus group display this psychological flexibility, understanding some aspects of life are now permanently different and therefore adapting their focus into manageable activities such as reading. This principle of acceptance of symptoms and acceptance of anticipation is fostered in acceptance and commitment therapy, which has shown promise as a psychological intervention for patients with chronic disease [27].

Emotional and psychological dimensions of DCM appear similar to those of other chronic diseases. Disruption of one’s lifestyle, career, and goals leads to protracted states of emotional distress, increasing the risk of psychiatric sequelae such as depression and anxiety [28]. Of note, the relationship between depression and anxiety and chronic disease may be interrelated as described above or independent, whereby the two conditions have separate origins but inevitably exacerbate each other [29]. Notwithstanding, the presence of psychiatric disturbances further exacerbates negative physical outcomes and increases disability, resulting in a cyclical disruption in psychosocial health [28].

Lack of life control is an established factor in the development of negative well-being states such as depression and anxiety [30-32]. Similarly, feelings of guilt by people with DCM with chronic disease can contribute to and worsen depression and anxiety [29]. In our focus group, the source of guilt was the perceived impact of the disability on the supporters of the participants. Indeed, in chronic illness, family members may suffer adverse psychological and emotional effects, such as worry, frustration, and stress [33]. It is important for the clinician to consider the mental health of the individual and their supporter network when managing people with DCM.

Finally, societal misconceptions about chronic diseases can have a significant impact on individual psychosocial health. Incorrect perceptions of one’s disease have been equated to a feeling of one’s reality being questioned—this reality including suffering not understood by others [34]. This societal stigma may adversely affect the doctor-patient relationship, as people with DCM are less open in anticipation of discrimination or stereotyping. Indeed, stigma and the anticipation of stigma results in lower treatment satisfaction and, more broadly, less social support and lower quality of life scores [35]. Raising awareness for DCM and promoting support groups for people with DCM (such as Myelopathy.org) are potential ways to address this.

Clearly, further investigation is required to confirm the determinants of quality of life in DCM, but the experience from this study and the broader literature is consistent. It is likely that a multidisciplinary approach to ameliorate the wide array of difficulties presented by DCM may be the answer in improving the QoL of people with DCM.

### Limitations

The selection of participants was based on pragmatic sampling—all participants were White, and the sample size is small (although this size is within acceptable ranges for focus group or style sessions) [36]. Importantly, the age of participants and their level of disease severity matched the average among high-quality clinical series [37]. There was also representation of nonoperative DCM (with one participant not yet having undergone surgery) and their close supporters. Additionally, the qualitative analysis style will be susceptible to variance depending on the researcher performing the analysis [38]. The interpretation and extraction of themes from the data are shaped by the researcher’s perspectives, personal biases, and style of language [38]. We attempted to address this with 2 independent analyses that were merged, and by using researchers independent to the original interview process. Finally, although supporters were engaged in initial parts of this qualitative process, the session highlighted above focused predominately on the lived experience of people with DCM. Further studies would ideally assess the differing views of supporters of people with DCM on the life impact of DCM.

While these aspects may limit the confidence for generalization of findings, in terms of the COS process, this will be mitigated via the involvement of a larger and broader people with DCM and supporter group and the presence of open questions for the first round of the Delphi survey. Specifically, the COS forms a component of the large, international multistakeholder initiative AO Spine RECODE-DCM [7]. Domains (categories of outcomes) will initially be agreed by the AO Spine RECODE-DCM management group, with reference to the Outcome Measures in Rheumatology framework, the literature [3], and the findings of this study. This will be used to categorize individual outcomes identified by people with DCM and the literature to form the first round of an internet Delphi survey. As mentioned, the survey will contain open questions to allow further suggestions for outcome measures not already identified. Consensus will be achieved via further survey rounds and a face-to-face consensus meeting. Digital technologies will be crucial in implementing the next steps of the Delphi process. Recruitment into the process will require wider advertisement through online health communities (eg, Myelopathy.org and AOspine.org), dissemination via computerized survey (eg, SurveyMonkey), and data processing via analytical software (Nvivo [QSR International] and Excel [Microsoft Corp]). Moreover, in an era where social distancing is paramount for safety, committee and consensus meetings will be reliant on online conference calls.

### Conclusions

This qualitative analysis of people with DCM perspectives has highlighted a number of prevailing themes currently unmeasured in clinical research or care. The determinants of low quality of life in DCM are currently unknown, and these findings provide a novel and so far, unique perspective. These will be used to inform the formation of a COS, in particular their domains, as part of AO Spine RECODE-DCM. The continued inclusion of online communities and use of targeted digital software will be essential to achieving a consensus-based core outcome set for DCM, which is inclusive of all relevant stakeholders, including people with DCM.

## Appendix

Multimedia Appendix 1 Consolidated Criteria for Reporting Qualitative Research checklist.

Multimedia Appendix 2 Topic guide.

Multimedia Appendix 3 Interview transcript.

## Abbreviations

AO Spine RECODE-DCM: Research Objectives and Common Data Elements for Degenerative Cervical Myelopathy
CAMHS: Child and Adolescent Mental Health Services
COREQ: Consolidated Criteria for Reporting Qualitative Research
COS: core outcome set
DCM: degenerative cervical myelopathy
DLA: Disability Living Allowance
DWP: Department for Work and Pensions
mJOA: modified Japanese Orthopaedic Association scale
NHS: National Health Service
NIHR: National Institute for Health Research
PCS: Physical Component Summary
PIP: Personal Independence Payment
QoL: quality of life
SF-36: Short Form Health Survey–36 item
