# Supplementary material for: Prevailing Outcome Themes Reported by People With Degenerative Cervical Myelopathy: Focus Group Study
Source: JMIR Form Res. 2021 Feb 3;5(2):e18732. doi: 10.2196/18732 (PMC7889422; doi:10.2196/18732)
Supplement: Multimedia Appendix 2 [file formative_v5i2e18732_app2.docx]

Supplementary Information

**Prevailing outcome themes reported by people with degenerative cervical myelopathy: findings from a focus group session.**

***Topic Guide:***

1. *Research goals of the focus groups:*
   1. Explore reported outcomes of DCM from people with lived experience
   2. Explore the impact of DCM of the lives of those with lived experience
   3. Identify common impact themes and explore perspectives of group on the same
2. *Introduction, overview of online activities - about 10 minutes*
   1. Welcome, introduction of researchers
   2. Introduction of focus group members: name, where from, DCM journey in brief
   3. Explaining of research goals
   4. Explaining of what an outcome is in the context of medical research and how this differs from the impact of the outcome. Therefore prior to starting, a group task was undertaken to list the potential effects of taking a train journey.
3. *Separate Supporter and Person with Cervical Myelopathy sessions – 20 minutes*
   1. Domains covered: Function, Pain, Quality of Life, mJOA sub-categories (Sensation, Walking, Bladder Function, Hand Strength)
   2. Allowing spontaneous discussion
   3. Taking notes of outcomes/impacts list identified during these sessions
4. *Exploring outcomes and their impact - about 40 minutes*
   1. Starting with an open question: “How does DCM affect you?”
   2. Allowing spontaneous discussion
   3. Re-orientating conversation to outcomes and the impact of outcomes (as per the lists generated from the initial sessions above)
   4. When an outcome or impact is identified, it will be written on card in large font and placed on a board for reference and to stimulate further discussion
   5. If the line of discussion is exhausted, we inquire about other possible outcomes/impacts in general:
      1. Does DCM affect work?
      2. Does DCM affect relationships?
      3. How else does DCM effect your life?
5. *Conclusion – about 10 minutes:*
   1. Summary of outcomes and impacts
   2. Thank you and close
