# Supplementary material for: Prevailing Outcome Themes Reported by People With Degenerative Cervical Myelopathy: Focus Group Study
Source: JMIR Form Res. 2021 Feb 3;5(2):e18732. doi: 10.2196/18732 (PMC7889422; doi:10.2196/18732)
Supplement: Multimedia Appendix 3 [file formative_v5i2e18732_app3.docx]

**Focus Group Transcript: “How does DCM affect you?”**

*Interviewer 1*: Right… so… so I think the first bit I just want to uhh.. I mean I find these discussions are useful with you bouncing each other/ off each other ‘cos you all forget/ have forgotten things and someone else prompts and you sort of remember. But I really just want to go through with you for 20 minutes how this (DCM) has affected you right from the beginning, if you think of the things that have affected you. What about you? [directed at group member]

*Group member 1*: Ummmm… I’m thinking… like uhh… I used to be quite active uhhh… I used to be active with work and active in the week as well so that one is uhhh.. I do what I had [unclear] I just stopped doing it.. my activities.. so…

*Interviewer 1*: Right... so what sort of activities would they have been?

*Group member 1*: I used to do martial arts three times a week, so that didn’t help the neck either…

*Interviewer 1*: Sure… sure

*Group member 1*: When I had to give that up, I knew it was something quite serious

*Interviewer 1:* So you lost your activities, how did that make you feel?

*Group member 1*: Yeah, that… that’s something that got me down because everyone else... all my mates are still drinkin’ and so on, so… it puts you y’know the things into a different perspective then

*Interviewer 1*: Yeah... yeah.

*Group member 1*: Your abilities... and again it’s tiring as well… so its [unclear] day by day. They come and say “ohhh we’re going to do that tomorrow” …

*Interviewer 1:* So, you’re losing your independence?

*Group member 1*: Yeah.

*Interviewer 1 1:* And social activities, they...

*Group member 1*: yeah… yeah…

*Interviewer 2:* Big letters because we’d like to put it on the floor. [Background noise]

*Interviewer 1*: anyone else want to share?

*Group member 2*: I think I would go along with that and even go further is sooo frustrating, because you cannot plan anything. You would not believe the organization that went in to getting me here today [background noise], but you... for one day to the next, you don’t know whether you’re going to be walking, staggering, whether you’re going to be, literally or more or less crawling around the house because you’ve had a fall or y’know….

*Interviewer 2:* Falls is another thing

*Group member 2:* You... you… it’s frustrating if extreme. You try to plan something, your child [unclear] I couldn’t tell ya how many things I…I… I’ve missed…

*Interviewer 2*: We need to slow down a bit I think [laughs] [background laughing]

*Group member 2*: So frustration…

*Interviewer 2*: Frustration… Falls you’ve mentioned… you said variability of symptoms.

Group member 2: Yeah, that was people’s perceptions, you talked about emmm… perception and being perceived by other people….

*Interviewer 2*: Yes… yeah.

*Group member 2*: Now this is something I really want to come through because a lot of people, and everybody around this table will know this frustration, that for all intents and purposes you look perfectly fine… [Group agreeing]

…Ya haven’t got a cast, ya haven’t got a… bandage on your head [yeah in background] … you look perfectly fine, you talk perfectly fine and all of a sudden y’ you’re on your back on the pavement and you can’t get up again.

*Interviewer 2*: Yeah, so..

*Group member 2*: And people think you’re drunk!!!

*Interviewer 2*: Getting up… getting up from the floor…

*Group member 2*: People think you’re off your face… Now generally people are very kind I have found in my… experience people are very kind. But that’s after they’ve kind of walked around and kind of given you the eye. So.. yeah frustration…

*Interviewer 2*: [noting down] Can’t get up

*Group member 2*: Loss of kind of independence, loss of physical…

*Interviewer 2*: Independence?

*Group member 2*: …physical abilities

*Interviewer 1*: And the social stigma that you were saying as well?

*Group member 2*: Social stigma… that’s… that’s…

*Interviewer 2*: then the variability of all this

[Background muttering: “uh huh”]

*Group member 1*: the thing I find as well is, if you’re nervous about something, the condition gets like 50x worse [background agreement]

*Background*: Anxious… anxiety

*Group member 1*: I’ve been there, I’ve gone to… say the co-op, gone to… uhh pay for my stuff, end up I dunno… my card, fumbling my card and then the shaking starts, and then it’s the dexterity and trying to do stuff... simple thing doin’ the shopping, doing the queuing?

*Group member 2*: The change [laughs]

*Group member 1*: the change is gone… yeah

*Group member 2*: [unclear] you can’t bend down to pick it up because you know if you get down there to pick it up you won’t be able to get back up again. Ummm… yeah sounds stupid…[mumbles]

*Group member 1*: you feel like you’re walking out with a… sort of a… a beacon on your head, with the way you move things and probably people don’t know this but I think as an individual yourself think hang on there, I’m going to have to do things differently

*[Background*: subconscious]

*Group member 3*: Anxious if you’re going to leave your own four walls [background agreement] you know that oooh I’ve gotta… travel… I’ve gotta do this and It gets your stomach all mmmm…

*Interviewer 2*: So the stomach? That means problems with your bladder?

*Group member 3*: Yes Yeah… yeah, bladder and bowel

[background noise, unclear]

*Interviewer 2*: pop that down as well then

Interviewer 1: what sort of problems do you have with your bladder and bowels?

*Group member 3*: You can’t control them properly…

*Group member 4:* When you’re going out you’re thinking where’s the toilets first of all or start planning

[Background agreement]

*Group member 3*: Yep.. I can tell you where all the toilets are in the town centre

[background comments from group member 4, unclear]

*Group member 4:* … I’ve suffered for a few days afterwards, I won’t be able to walk but I’m going… where’s my toilet now or I’m in real trouble.

*Group member 3*: you’ve gotta know where there’s toilets

[background group member 3: bowel control is…]

*Group member 2*: You have to be very careful of what you eat… don’t you?

[background agreement]

*Group member 3* [at the same time as group member 2]: yeah bowel control you need to be within minutes

*Group member 2*: have to be careful of what you eat, where you eat, if you’re going out, how long… [background agreement]…before you go out you have to [unclear] carefully.

*Group member 2*: you know this is not a very nice subject so let’s get it done and dusted and out of the way. Personally, I take some medication… every morning and then ammm… just kind of make sure ammm… because I had a situation where I have been caught and it is the most embarrassing thing in the world.

*Interviewer 2*: Of course, yeah

*Group member 2*: Ammm… so there we go, that is the most horrible aspect of it, well socially, yeah horrible aspect of it

*Group member 1*: I know, the thing is, it’s emptying the bladder as well, you know, I go to the toilet and then within 10 minutes you’re back in the toilet

*Interviewer 2*: You’re back again… yeah

*Group member 1*: and you can’t, especially in the morning if you get up/it

*Group member 4*: Which they told me was kidney stones, they said y’know because I’ve had kidney stones 20 years ago, they said uh it’s just kidney stones, that’s all it is y’know you need to keep going…

*Interviewer 2:* so difficulties emp… emp emptying the bladder

*Group member 4:* Yep

*Interviewer 2:* increased frequency… I think putting on different…

*Background*: Control…

*Interviewer 2:* Control yeah.

*Group member 5*: For me mines a physical thing because of the reduced dexterity it’s hard to get your clothes down in time [background agreement] if one of you could write this down while I’m speaking?

[*Background:* yeah]

*Group member 5*: Ummm… Also physically getting to the toilet, if there’s any trip hazards there and I’m tryin’ to negotiate trip hazards, I’ll wet myself because it delays me. If the kids… I’m a lone parent… and they’re all boys… leave the toilet seat up or if they’ve left no toilet tissue, I know it’s the simple things like that! And am thinkin’ I can’t get there in time and y’ start to panic and y’know ‘cos I…

*Background:* it’s a long plan isn’t it

*Group member 5:* And obviously I [unclear] using incontinence products. I’m not proud of it but that’s what I have to do!

[Background agreement]

*Background female:* I do too

*Group member 5:* Y’know so… and and [unclear]

*Interviewer 2:* So incontinence is another one

*Group member 5:* Yeah I’ve got that one and also this is really gross. Am sorry chaps but I’m still a menstruating woman, when the pain and dexterity is bad, I struggle to use tampons [background agreement ‘Mmm’] and things like that, because I can’t reach round properly. And I know it’s horrible to talk about but…

*Interviewer 2:* No I think we should…

[Background agreement]

*Group member 5:* Women’s issues don’t always get…

*Interviewer 1:* Let’s talk about that dexterity bit because that’s obviously another issue isn’t it?

*Group member 5:* I might have put down already my number 2…

*Group member 4:* Lack of sleep I find one of the worst,

*Background:* YES

*Group member 4:* that burning at night, problems are always worst at night

*Group member 5:* Restless legs

[background group member: going into spasm]

*Interviewer 2*: There’s so much we need to ah… [laughs]. We need to sort of slow down a bit otherwise we…

*Group member 5:* I can’t keep up ‘cos [unclear] in me [unclear]

Background laugh

*Group member 1*: my legs were going last night

*Group member 5*: Restless legs ohhh gosh…

*Group member 2:* Are we all taking sleep medication?

[background agreement]

*Group member 5*: N… I just take co-codamol and I prefer not to take anything stronger

[background noise unclear]

*Group member 4* [at the same time as group member 5]: They don’t like givin’ it ya to be honest, it’s bad pain management

Background conversation

*Group member 4:* [start of sentence unclear] It all starts in the left leg and then suddenly it’s in been in the hands, up the arms, and it’s pain

*Group member 4:* and It’s spreading.

[*Background*: Burning! Burning.]

*Interviewer 2*: So burning pains? In legs and arms

*Group member 4:* It spreads, even in your face

*Group member 5:* Yeah, and that’s the other thing I got told ‘oooh XXX(ppts name), It’s in your neck, it shouldn’t be in your face. But I get… I get itches, itchy scalp, an itchy face… and ticks and tip of my nose

*Interviewer 1 1*: and I know you get headaches as well

*Interviewer 2:* So, the headaches… the headaches there’s an explanation because basically you get cramps in your muscles around the neck but the other things are difficult to understand

*Group member 4*: Breathing as well, I get a bit like [unclear] When you lie… when I lie down, I have asthma… as soon as I lie down [makes sound] it’s struggling to breathe.

*Interviewer 2*: So that’s an extra card

*Background:*  Alright, I’m getting there

*Interviewer 2*: Yeah, I know, sorry. If you need some help

*Group member 5: [writin]* Breathing? Uhhhh

*Group member 4*: from directly above: if I sit up…

*Interviewer 2:* Yeah?

*Group member 4*: If I sit up, it’s when lying down especially on the back flat you literally struggling to breath.

*Group member 5:* Yeah, I’ve got a cough, I’ve had a cough for years.

*Interviewer 2*: So, so everyone has noticed problems with their breathing?

*Group member* 6: Lying down definitely…

*Group member 1*: Yeah, it’s like a… [unclear] … Yes [unclear] A little bit of a rattling in your chest

*Group member 5:* YES In the morning when you get up… yeah… yeah

*Group member 1:* [unclear] if you lie down you get it again… yeah

*Group member 5*: I’ve resorted to a ventolin inhaler before I go to clear my lungs in the mornings

*Group member 2:* Well, I’ve sort of e… end up…

*Group member 4*: As soon as you lie down it starts.

*Interviewer 2*: It starts?

*Group member 2*: … I’m sleeping on sort of four pillows, so I’m sort of sleeping in that position

*Interviewer 2*: So you’re propping yourself up?

[Background agreement]

*Group member 5*: See I have to have it right down… I have one pillow if I can, [unclear] an alignment, for the minute… my neck pain basically

*Interviewer 2:* Ok…yeah

*Group member 1:* I’m lying flat sometimes and… I think if I’m lying on the bed for about three nights, I’m having lower back problems, so … [background agreement] I’ve only had that since surgery and didn’t have no problems with my lower back until surgery. And… when I came round… amm I was in surgery for about 5 and a half hours so I came out [unclear] and when I lie down… I couldn’t lie down at all [unclear] and it’s carried the problem through as well because as I said on my mobility chair [unclear]. So, then I had to go back to bed [unclear] 2 nights from the lower back pain again, and it’s so severe that it wakes you up.

[*Background:* Mmmm]

*Interviewer 2*: So lower back pain is bad…

*Group member 4* : yeah yeah that stiffness

*Interviewer 2:* Stiffness!

[*Background female member:* because I… it’s…. [unclear]]

*Group member 4* : well pain is [unclear] If I slightly overdo it at the weekend, I said I’d out and watch the rugby, just staying in there. The next 2 days, I’ll not be able to walk properly for 2 days and then it’ll ease off, then I can get mobile again.

*Interviewer 2:* It’s like you’re punished for doing…

*Group member 4 :* I expect that… yeah.

*Group member 5 :* If you don’t pace yourself properly… yeah

*Group member 4*: They tell you about pacing, but you’ve got to go out and do sommit’, so I can’t do… Exactly! ….

*Interviewer 2:*  Otherwise you’re suffering for 2 days. if you’re withdrawn… [unclear]

Group member 4: …Get outside and you don’t wanna do that so…

*Group member 4* : I expect the two days of pain, I know it’s coming, I know I’m not going to be mobile for two days but I just live with it.

*Group member 5* : Alright, tomorra I’m going to be doin’ F all

[Background laughing]

*Group member 2 :* It’s a vicious circle though isn’t it? Because, if… if you… when you go, and we have to make ourselves go out and do things or otherwise you just… y’know. But then it’s a vicious cycle isn’t it? [Background agreement] Because you go and do it, ya either have an incident when you’re out, or… as you say you’re suffering with your back the day or two afterward and then you get anxious about doing that again because you think you about

*Group member 4 :* I say I’m doin’ it, and that’s what I like doing, ‘cos I know I’m gonna suffer [unclear] I’ll just do it, I’m just gonna carry on doin’ it because I know I’m going to suffer but I just accept it.

*Background*: Mmmmmm

*Group member 5 :* And from my perspective when I found out that ummm… I was glad that I had the surgery don’t get me wrong because it was halting progression, because you’re damned if you do and damned if you don’t, so I thought I’ll have the surgery …ummmm but when they told me that I wasn’t going to get any better, as I said I was devastated and I actually ended up feeling suicidal [background mmmm]. Because as a lone parent I’ve got to do for my family, my boys are older now it’s a bit easier, but they still don’t fully understand and I still have to do everything because they’re boys [background laughing] sorry that’s sexist! I’ve got a 20 year old an 18 year old and an 11 year old and it’s had a massive impact on my 11 year old, he’s … [handed something] thank you… he’s currently going through CAMHS at the moment because he’s struggling ‘cos he’s got now two disabled parents. And it’s simple things like being able to take my son to the park, I might get upset now.

*Interviewer 2*: I understand

*Group member 5:* Going on holiday, I mean I was in agony walking up my auntie’s camp… it’s now… it’s like It’s put such restrictions on [background agreement]. Sorry I’m going on a bit.

*Group member 2*: No no, you’re not because this is a huge part of your life…

*Group member 5*: And it’s ummm…

*Group member 6??* : It’s the simple things you could do like walking, I used to walk [unclear] 3 miles [unclear] I don’t go halfway now, I’ve been in crippling pain halfway down the footpath.

*Group member 2*: But XXX (ppt 5’s name), this is going to have a profound effect on her relationships with the boys…

Group member 5: Well to be honest I’ve been on anti-depressants for the past two and a half years and there was never really a good time to come off them.

*Interviewer 2*: Can you write down depression?

*Group member*  : Yeah, I will do.

*Background* : Because that’s a big part

*Group member 2: I* think this is wider thing effects our families, emm because they’ve obviously grown with the knowledge of the disease with us as we’ve had it and we’ve [unclear]

*Group member 5*: Guilt yes it’s a big one! I feel guilty…

[everybody talking at once, unclear for a few seconds]

*Group member 6:* [unclear]and now I have to watch. They were wrestling with me [unclear] leave it alone

*Group member 3 :* I mean my children have just left home but yeah guilty for them because they see me crippled up and in a mess

Group member 4 : You feel like a lame duck doing basic stuff that you know physically you could do

[Group member 6 agrees].

*Interviewer 2*: Feeling like a lame duck?

*Group member 5*: A lame duck… Yeah.

[Background agreement]

*Group member 4:* With simple thing like lifting bags of shopping.

*Group member 5:* Well we’re not supposed to lift are we?... I still do stuff that I’m not supposed to do because there’s nobody else to do it [laughs], you have to carry on and then you get the DWP saying that ‘oooh you’re not disabled, you can do this you can do that’… I … I mean I’ve lost my DLA, I’ve been given zero points for PIP, I’ve lost my Motorbility Car, I’ve had to buy it, I’ve had to go into more debt than I’m already in to get… buy my car. I’ve lost my blue badge! Even a simple thing like a blue badge cos’ the council won’t accept that I can’t do… I can… yes it looks like I can walk more than 20 metres or more than 50 metres, I can’t do it reliably, I can’t do it repeatedly, I can’t do it with increased pain. The fatigue! The fatigue is the one that gets me, I go to bed for three or four hours everyday, I’ll take the kids to school, walk the dog and I use the term ‘walking’ loosely.

[*Background:* that’s alright]

*Group member 5:* I use the terms loosely because I walk where I can let the dog off so she can do a run and she can do everything. I have to focus on absolutely every step, even the texture of the floor is enough because I get hypersensitive feet. I either feel like I’m sticking to the floor, or I’m walking through treacle, or the actual…if there’s any gritty bits on the floor it’s a bit like… I call it princess and the pea syndrome, it’s like one little gritty bit is enough to make you stumble, do you know what I mean? Ummmmm, but yeah.

*Group member 1*: I…I find sometimes when I walk barefoot in the house, it feels like there’s a bit of plastic stuck…

*Group member 5:* My feet go into spasms.

*Group member 1:* Yeah…I am like that

*Group member 3:* Escalators I can’t do now because they send you into a wobble.

*Group member 5:* Yeah, they do send you into a wobble, you’re right and if there’s a lift I take it but there’s not always a lift, which makes…

*Group member 3:* I don’t like lifts but I’d rather have a lift than an escalator now, because you go...

*Group member 1:* Yeah it’s a big problem now disability wise, they don’t recognise it do they?

[Background: No]

*Group member 1:* I had to fight for mine as well because in 2014 they wouldn’t give me disability at all, because I hadn’t had surgery or anything and they…they… filled in the papers, they sent it back saying ‘no you can’t have it’.

*Group member 2:* I think it’s post code lottery…

[Background: It is, it is]

*Group member 2:* [unclear]… only before, the lady that did my ESA assessment the second time, she had a sister in law who suffered from cervical myelopathy, so the minute she saw my form she knew exactly what I was talking about and I was fine. And similarly, when I went to have my PIP done which I understand they didn’t give you and she… my son came with me, and as soon as I started talking to her about bowel incontinence she said ‘okay fine, that’s all we need to know, you can’t be expected to go to work like that’

*Group member 5*: No, well I won my first ESA on the tribunal, and I got… and I got… and I go fifteen pass. And I’m just been... that was about three years ago, I’ve just been reassessed for ESA, I’m in the support group but as I say it’s took tribunal to get there, ummm… I’ve just been reassessed so I’m dreading the envelope at the minute [laughs]

*Group member 2*: The thing is it’s a lottery!... It’s a lottery.

*Group member 5*: It is but… as… as with DLA, I’m on high rate mobility and medium rate care! So I was a danger to myself at that point and although my mood has stabilised, I’m still at risk of falls and things.

[Background agreement]

*Group member 5:* And now, I’ve been to… my tribunal has been postponed because they want more evidence from the DWP and I’m hoping there isn’t any trouble because there’s inconsistencies in the medical report, and I have to wait for a new tribunal date so I’ve already been waiting six/seven months now and I’m yet to go back.

*Group member 2*: But this is what we’re talking back to frustration…

*Group member 5*: Yeah…

*Group member 2:* … and planning, because okay we don’t want to get side-tracked in the DWP because we could go on forever…

*Group member 5:* Oh we could! I don’t mean to bring it up but…

*Group member 2*: … but It goes back to this same issue of not being able to plan because it’s a lack of… if you’re not confident in your finances and what you can provide for your family and that’s a frustration in itself and it’s going to make you depressed and angry and down, and that in itself is not good for your general wholesome health.

*Interviewer 2:* It’s an impact on… on you… you’re ability to earn to…

*Group member 1*: It’s like a domino effect

[Background agreement]

*Group member 2*: You can’t plan! This is the central core of all these issues, be it finance, family, future, your social circle, you can not plan. There’s no consistency [background agreement], one day you can walk, one day you can’t, one day you can travel, one day you can’t. You cannot always rely on having, I mean my sons are grown up young men, they have girlfriends, they have careers, they have lives, I cannot be saying to them ‘sorry kids, today is a bad mum leg day, can someone come and take me from a…

*Interviewer 1:* Right, let’s trace that back a bit because obviously the core needs is the planning, but what’s stopping you from being able to plan?

*Group member 2:* Because there is no consistency with my mobility.

[Background agreement]

Group member 3: You don’t know until you wake up, whether you’ll be able to walk or not.

Group member 5/6?? (male): you don’t know if you’re going to sleep at night do ya? You may have an appointment tomora. For instance I was still wide awake 3 in the morning.

Interviewer 1 OR 2: [unclear]

*Group member 2:* You miss social engagements, you miss… there is no part of your life that you can say I will definitely be at point A tomorrow or point B tomorrow because you just don’t know.

*Interviewer 1:* I’m just going to draw you down a couple of points, if we go back to mobility issues, so what for you… what is the core mobility problems

*Group member 2*: The core mobility is my gait and my balance

*Interviewer 1 and 2:* Balance, perfect!

*Group member 2*: If I… I can wake up in the morning and the minute I put my legs up beside the bed I know ‘SHIT’ this is a wobbly day.

*Interviewer 1:* Okay

*Interviewer 2:* Yeah

*Group member 2*: Now I cannot stay in the house all day, wobbly day or not, there are going to be thing I have to do. So, I either get out there and do them and I run the risk of a fall, which makes me anxious, blood pressure goes up and you’re shaking and that makes simple things difficult. So what’s you’re other option, housebound?...

*Group member 5:* Stay in the house

*Group member 2:* … Tesco online?

*Group member 5:* That’s what I have to do.

[Background agreement]

*Group member 2:* That’s your options.

*Group member 3*: Rely on your carer. You rely on you husband, wide, carer [background agreement]. They do so much.

*Group member 2:* You see I live on my own, so I don’t have that backup support.

*Background:* No.

*Group member 5:* No same here.

*Interviewer 1*: Just getting back to business, is it all the same with balance?

*Group member 3:* Yep.

Group member 5: Balance and pain

*Group member 4*: The thing is with the balance is… sort of the legs just go rigid and just don’t work…

*Group member 5*: And sometimes my knees actually give…

*Group member 4*:… And then you start stumbling…

*Interviewer 2*: Less control?

[Background agreement]

*Group member 4*: Yeah! You start off alright and then the further you go the burning will start and then you start dragging your legs and sort of…

*Group member 2*: It’s like a marionette y’know.

*Group member 1*: [unclear]

*Group member 6:* Yeah I start off alright and get slower and slower and get more and more I am struggling

[unclear for a few seconds]

*Group member 6:* The further I’d go the more id struggle.

[Background agreement]

*Group member 5*: But my legs actually give way, especially from trying to get up and down the stairs as well, spontaneously without warning and it can be in my both ankles and it’s always my left knee, it always gives way.

*Interviewer 2*: [unclear]…. Increase as you walk?

*Group member 4:* Also the other thing on that, then when you’re walking funny it’s hurting your hip, I dunno if anyone else feels that and You feel pain then from other areas.

*Group member 3*: Hips, knees, ankles.

*Group member 1*: Achilles I find.

*Group member 5*: Yeah, I get real tight spasms I find

*Group member 1*: I tore my achilles a few years and thinking back now, could it be a result of what I actually have… so.

*Group member 5:* And also if you use walking aids like we do, us three’ns here, you’re putting more wear and tear on the arm or the other arm if you use both, yeah you put more wear and tear on there as well.

*Interviewer 2*: Yes, tell me about the more general problems that you have. I mean wear and tear, this is not only confined to the neck, often it’s the knees as well, the ankles, the lower back

*Group member 5:* … Hips, I’ve got sacroiliac joint disease as well. But again, I get told it’s because I’m fat! But I got fat because I became immobile, not the other way around. Y’know? And talking about goin’… mobility… I when I was diagnos… when I found out I wasn’t getting better, I actually did not go out unless I had to, I was in the house for about a year. Apart from taking the kids to school, I actually got a dog.

*Group member 2*: [unclear] empathise that’s no good for you

Group member 5: No it’s not. I got a dog to get me out and that’s the reason I go out, not because I have… I should do, but because I have to. I have to obviously with having depression I really struggle to motivate myself.

*Group member 2*: But you see, who wouldn’t be depressed if you find yourself, and this is what terrifies me particularly because I live by myself, this is what terrifies me, the fact that I’m going to end up y’know with Tesco online sort of bringing me everything I need and then I’d be so afraid to go out of the house, you’re going to end up clinically depressed [background agreement]. I mean thankfully, I don’t know anyone who doesn’t leave the house with cervical myelopathy, I think you would all agree with me, some form of depression. But I don’t want to… I’m very reluctant to start taking anti-depressant tablets, I don… I….I force myself to go out, even if It’s to only go out to the shop and back, but it’s that… the not being able to know, it’s the frustration, not knowing in the morning, am I going to be able to

Group member 3: We have all got Myelopathy and everyone’s on a different concoction of drugs?

*Group member 5:* Well I only take over the counter medication because I was offered Gabapentin. I don’t really fancy Gabapentin ummm because I really do need to keep my wits about me, I drive, I’m the only person in the family who does drive, I’ve got the kids to look after, I’ve got dog and bunnies to look after, I’ve got 6 bunnies as well, again because I don’t get out much [laughs]. And so it’s… it’s really important for me to keep m…as clear as I can, and some days I’m quite forgetful ummm… I do get foggy in the old head.

*Group member 4*: I find that because of the medication [unclear] Concentration [unclear] I don’t drive much now because of the medication because I’m groggy, I know I’m not concentrating, and you make mistakes. Y’know, I left the grill on, turned the microwave, cooking summin [unclear] It’s happened three or four times, just totally forget! Normally I wouldn’t forget anything, it’s just from lack of concentration…

*Interviewer 2:* So, side effects of the medication

*Group member 1*: Social events like, so Ben said are you comin’ down and I said it depends on the day because if I was really bad I wouldn’t be able [unclear] because I wouldn’t be able to take my medication. If I haven’t taken my medication then I come here and I am shaking [unclear]

*Interviewer 2*: So shaking, everyone has shakes or tremors?

*Group member 5*: Ummm, to some degree

*Group member 4:* the legs don’t really but the hands do

*Interviewer 2:* above the hands?

*Group member 3:* Spasms.

*Group member 5:* It’s more stiff, I feel like I’ve got really fat fingers…

[Background laughing]

*Group member 6*: You can’t grip anything…

*Group member 5*: I can grip but it feels different.

[unclear for a few seconds, everyone talking at once]

*Group member 3:* My wife has to dress me if I’m putting a shirt on

[unclear for a few seconds, everyone talking at once]

Group member 2: … With knives and bowls… burning yourself [unclear]

*Group* member 3:… jam jar lids, stupid things

*Group member 6:* Things you could do before

*Group member 2:…* you forget, because you can’t feel it, doing things like change or umm… trying to… I mean [laughs] I was trying to do uhhh the laundrette the other day and trying to get the… coins… into the machine, I couldn’t…

*[Background:* fit them in]

*Group member 5*: I got timed out of the parking machine for that because I didn’t get the coins in in time, I had to start all over again it’s like wahhh.

*Group member 2:* Even stupid things like trying to get your cash card into that machine and fourrrr [unclear] [mimicking typing]

*Group member 5:* Yes! I’ve been timed out before on that one

*Group member 2*: …Trying to get your pin number in and everyone’s looking at ya as if you’re, y’know, you’ve got the worst case of DTs

*Group member 1*: The thing is when you do that you shake more then

[Background agreement]

[unclear for a few seconds, everyone talking at once]

[*Background*: you start rushing…]

*Group member 4:* … you make more mistakes

*Group member 5:* And going back to nitty gritty stuff, sorry guys I mean I’ve not been in a relationship in ten years but I imagine that sometimes having a fulfilling sex life might be quite difficult for people.

Background agreement

*Group member 3:* It is difficult

*Group member 4*: Yep, erectile dysfunction

*Group member 1:* My wife [unclear] my neck clicked and she goes ‘uhh did you hear that click’.

*Group member 4:* Another thing I [unclear].. Temperature. I’m hot all the time…

*Group member 5:* Yes! But I’m menopausing

*Group member 4*: Particularly in the house, I’m sweating all the time, especially at night, dripping with sweat. I’ve got the window open, I’m not wearing a shirt or anything, I’m lying on top of the bed, dripping with sweat, and then in the house… cold clammy sweats and then I’ll say ‘it’s cold, it’s freezing, I’m boiling’ but I’m effecting other people, they be saying ‘don’t be putting the heating on’ or anything like this or ‘leave the door open, let the air in’ … you’ll effect other people.

*Group member* *1*: But in the winter as well, I noticed it can be worse in the winter ‘cos my hands [unclear]

*Group member 5*: Sitting for fifteen minutes, I’ll stiffen up, standing up for like more than fifteen minutes I start to get back pain and my legs start to burn so I constantly have to change position usually.

*Group* member 2: Standing still is actually one of the worst things… yeah

*Group member 5* [simultaneous to group member 2]: It’s agony! It’s easy to walk than it is to stand still if that makes sense

*Interviewer 2*: Standing still.

*Group member 5*: uhhhh

*Group member 3*: My daughter gave me a fan, just an old-fashioned fan just to sit there… [Background: really]…and it’s silly but it works!

*Group member 5*: And another thing that’s frustrating, you go to your gp, I’m a… I’m a woman… I’m fo… [laughs] as you can see… ummm I’m forty-eight now so I’m g… I’m hitting perimenopause sort of thing and they sort of like say ‘ohhh it’s your hormones’ or or because I’ve got depression, I’m always constantly worried that it’s all in my head, and it’s not all in my head…

*Background:* No

*Group member 5*:… There’s a physical cause, it’s like… you feel like banging your head against a brick wall trying to get people to understand you.

*Group member 2:* I think we could sum it up in three things here. The frustration, uhhh frustration, depression and physical infirmity is really what we’re talking about here, I mean we’re physically infirmed, were not as physically fit as we used to be. I think I… speak for all of us. Here, all look perfectly healthy on the outside, I was a very fit person, I was a dancer for many years…

*Group member 7*: Oh that must be heartbreaking.

*Group member 2*:… in fact I thought initially that this might be… now I mean I wasn’t dancing in killer heels like they do on strictly, but certainly they were three or four inches, and I danced from the time I was about ten until I was twenty five and I thought this is classic ummm… high heels syndrome was what I initially thought when they said you have spinal damage. But I also gardened, I swam, I walked, I had two very active boys, I played mini rugby with them for years

*Group member 3:* Kicking the ball about.

*Group member 2:* Y’know I was a very physically active person, and it’s very difficult this part of the depressive cycle because when you… part of physical activity makes you feel good, it gives you endorphins, exercise is good for you, it’s not just good for you physically, its good for you mentally. And when you can’t do that physical exercise because you physically not fit enough, there’s a big gap in your life… what do you fill it with?...

*Group member 5* [simultaneously]: it’s a bereavement

*Group member 2*:… I… I have become… I am y’know I must be the library’s best customer. I… I’m in and out constantly doing six… now I did learn to speed read at university but even so, I’ve gone through six or seven books a week.

*Group member 5*: Wow I can’t even read that long now.

*Background male*: Are you all still working?

*Group member 4::* I’m officially still working, but were going down the ill health retirement, it’s gonna be sorted in the next three months… so I’m probably finishing in the next three months.

*Interviewer 2:* So one thing that I’ve ahh…feel that we’ve not discussed yet is… how do you… how do you experience the symptoms worsening over time, and how… how I mean… what happened first, how did you?

*Group member 4::* Well they say myelopathy is… I don’t know if people they had neck trouble did they?

[Background agreement]

*Group member 4:* … Well I have no neck trouble! And I’ve researched it and everyone starts with neck or pins and needles. I had none of that, all I had was the legs! Just heavy, couldn’t walk properly.

*Background:* mmmm

*Group member 4:* And when you’re back, Because the white dot on the board, I think this has been going on for years, and there were spells, only short spells, I just walk round the site at work and your legs are heavy. So, I’m assuming this was going on for about five years that’s why I got the [unclear] I had no pins and needles, no neck pain, nothing. And then couldn’t turn in bed, it started to get worse, so I said I dunno. Went to the doctor, said the neck was not working. So he said no, we checked the veins, y’know we think it’s going to be… you know problem-wise the muscles and whatever they call that. He said no you’re fine [under breath: I don’t know]. No the thing that convinced me, I went to turn the clock radio on to go to work, put my hand out, and It’s stopped, it’s froze…

*Group member 5:* Yeah.

*Group member 4:*:… for about seven seconds. I said, the hells goin’ on… and then suddenly went and I thought oh hold on, nah there’s something seriously wrong. That totally scared me, I thought what the hell is going on here, I can’t switch the radio on, I go more than for about seven seconds. So I went back to him and says again, the legs, I can’t walk and now I’ve had this, there must be something there. So he sent me to someone, and they were checking for more Parkinson’s and various. And they did the old ummm… they send you… the x-ray of your brain and neck sort of. I said ‘what’s wrong with me neck’ [unclear] ‘That’s not me neck, what you x-raying my neck for, It’s my legs is what’s wrong’. Anyway, because you have to wait for the scan for a while so I thought ‘im sick of this’, so I paid to go and get it done and said uh ‘can I pay for the scan, just to get it through quicker’ and they said yeah come tomorrow [background laughs]. Next day you can come quarter to two, I went to this place, lovely and relaxed, no stress, in this cabin, had the scan, I thought you go away and get results. He called me in within twenty minutes. What do you call it… radiology? He could read it, he said ‘because this brain thing I know you’ve been worried, I’ll tell you, your brains okay’ yeah, great. ‘But I’ll tell you what your problem is, it’s showing me the disks, the white dot on the spinal cord’, now he said ‘this is serious’. He goes ‘I know your consultant, do you want me to phone him?’. Fair play, phones him up, [Background: that’s lucky] explained the process. He said you have to be passed onto a neurosurgeon… I was lucky, ‘cos I paid for it. The neurosurgeon, he says ‘I can’t repair you, I’m not a plumber’ he said. I thought hey? You’re going to cut me throat and take my disc out and you can’t repair it? I phoned [unclear], he said I said Im screwed here. He can’t repair me, just got to stop me getting worse. It sorta shocked when he said he said ‘I can’t repair ya’.

*Interviewer 1:* Do you think things have changed since you had the surgery much?

*Group member 4::* My symptoms! And the burning, well I’ve got this burning and I didn’t have… [unclear] first my hands we’re burning, sitting in a pub with my son, both hands were on fire… I thought…. I think this was two weeks before the operation…

*Interviewer 2 & Interviewer 1*: what else?

*Group member 4:…*but the burning carried on. And the burnings got worse, so the turning in bed it cured that, but the burning came since the op…

*Interviewer 1:* Right…

*Group member 4:*…and it’s there all the time now so, they’re increasing the medication next week the amitriptyline. I’ve tried them all duloxetine. Im on amitriptyline anyway but sort of umm adjust pain management but also stick with the amitriptyline.

*Interviewer 1:* Just wondering, anything else we haven’t covered?

*Interviewer 2:* I…I… have we covered everything? …Y’know we can’t cover everything, but are we happy with what…

*Interviewer 1:* I just wondered if we start tryin’ to go back through everything, to see if it triggers anything were kind of missed out. I mean just trying… we did try and sort of group them a little bit…

*Interviewer 2*: Well we’ll do that once we have uhhh… everyone back.

*Interviewer 1:* Yeah, and do it together?

*Interviewer 2*: So I think uhhh, let’s uhhh… if we’re happy we’ll just have a quick break, and we’ll get uhh…the cards then and we’ll go

*Group member 4*: Are you happy to be called carers? You haven’t got a better term for [unclear]

*Group member 5:* Supporters.

[Background agreement]

*Group member 2:* Family…

*Group member 5*: But what I’m about to say, I mean ummm, just purely, I think it’s so important that your assessment does include some sort of qualitative aspect because it does affect us qualitatively. We’re not just numbers on a page. I mean I know because I find that the numbers don’t… not when you’re ticking boxes like the neck disability index and stuff like that. Well you think, I ain’t got any neck pain, but yeah and it…it was really difficult to actually fill in the assessment tools. Soooo…

*Interviewer 1:* So… you don’t think they’re applicable to you?

*Group member 5:* Well in certain case-s because obviously on the neck disability index they do ask you about your sex life and I thought ‘well none’ [laughs] you know what you do… embarrassing! Y’know uhhhh, sort of thing, y’know.

*Group member 2:* What you should do is write big letters, I… I… next time, I’ll find out!

[Background laughing]

*Group member 5:* I like that

*Interviewer 1:* Excellent! Well hopefully, the whole ambition is that by trying to work out what…how you’re being affected we can try and…
